# Supplementary material for: Aflatoxin B1 Metabolism of Reared Alphitobius diaperinus in Different Life-Stages
Source: Insects. 2022 Apr 6;13(4):357. doi: 10.3390/insects13040357 (PMC9025786; doi:10.3390/insects13040357)
Supplement: Supplementary file 1 [file insects-13-00357-s001.zip › insects-1650205-supplementary.pdf]

Table S1: Instrumental MS/MS parameters of mycotoxins analysed in positive ionisation mode.

**Component**	**Q1**	**Q3**	**Dwell (ms)**	**CE**	**CXP**	**DP**
Aflatoxin B_1 (ql)	313.000	128.100	5.0	91.000	10.000	40.000
Aflatoxin B_1 (qn)	313.000	285.200	5.0	33.000	16.000	40.000
Aflatoxin B_1 (ql3)	313.000	241.000	5.0	54.000	15.000	40.000
Aflatoxin B_1 (ql4)	313.000	213.100	5.0	63.000	15.000	40.000
Aflatoxicol (qn)	297.100	269.000	5.0	29.000	16.000	66.000
Aflatoxicol (ql)	297.100	114.900	5.0	81.000	12.000	66.000
Aflatoxicol (ql2)	297.100	141.000	5.0	65.000	14.000	66.000
Aflatoxin M_1 (qn)	328.900	272.900	5.0	33.000	18.000	61.000
Aflatoxin M_1 (ql)	328.900	229.000	5.0	55.000	16.000	61.000
Aflatoxin P_1 (qn)	299.000	271.000	5.0	33.000	18.000	101.000
Aflatoxin P_1 (ql)	299.000	114.900	5.0	71.000	12.000	101.000
Aflatoxin P_1 (ql2)	299.000	90.900	5.0	67.000	10.000	101.000
Aflatoxin Q_1 (qn)	329.100	310.800	10.0	29.000	16.000	71.000
Aflatoxin Q_1 (ql)	329.100	177.000	10.0	45.000	22.000	71.000
Aflatoxin Q_1 (ql2)	329.100	128.000	10.0	67.000	14.000	71.000
IS Caffeine ^{13}C pos	198.000	140.000	5.0	25.000	11.000	71.000

Q1: First quadrupole; Q3: Third quadrupole; CE: Collision energy; CXP: Cell exit potential; DP: Declustering potential.

Table S2: Recovery percentages of analysed compounds.

**Compound**	**Frass D14**	**Larvae D15**	**Frass D27**	**Insects D28**	**Frass D48**	**Insects D49**
AFB_1	102.6	75.6	94.7	72.2	96.5	74.4
Aflatoxicol	120.3	71.9	88.4	80.0	96.1	85.0
AFM_1	92.9	72.6	90.2	79.6	87.8	73.5
AFP_1	107.1	93.9	115.9	107.2	117.7	93.8
AFQ_1	93.9	77.1	123.8	87.8	88.3	83.3

AFB_1: aflatoxin B_1, AFM_1: aflatoxin M_1, AFP_1: aflatoxin P_1, AFQ_1: aflatoxin Q_1

Table S3: Concentrations of n=10 samples analysed with LC-MS/MS in duplicate to determine homogeneity of substrate.

**Replicate number**	**200 µg/kg**		**600 µg/kg**	
	**Duplicate 1**	**Duplicate 2**	**Duplicate 1**	**Duplicate 2**
1	181.3	188.7	567.4	570.4
2	193.0	190.1	549.5	563.4
3	192.2	190.8	569.4	545.8
4	190.3	192.2	566.3	552.5
5	193.7	191.2	560.2	555.3
6	184.5	184.6	548.6	553.3
7	185.8	188.5	557.9	555.0
8	184.7	180.8	546.9	561.8
9	181.4	188.6	545.4	563.4
10	189.5	187.6	552.2	560.7
